# Overexpression of plastidial thioredoxins f and m differentially alters photosynthetic activity and response to oxidative stress in tobacco plants

**DOI:** 10.3389/fpls.2013.00390

**Published:** 2013-10-16

**Authors:** Pascal Rey, Ruth Sanz-Barrio, Gilles Innocenti, Brigitte Ksas, Agathe Courteille, Dominique Rumeau, Emmanuelle Issakidis-Bourguet, Inmaculada Farran

**Affiliations:** ^1^Laboratoire d’Ecophysiologie Moléculaire des Plantes, Institut de Biologie Environnementale et Biotechnologie, Direction des Sciences du Vivant, Commissariat à l’Energie AtomiqueSaint-Paul-lez-Durance, France; ^2^UMR 7265 Service de Biologie Végétale et de Microbiologie Environnementales, Centre National de la Recherche ScientifiqueSaint-Paul-lez-Durance, France; ^3^Aix-Marseille Université Saint-Paul-lez-Durance, France; ^4^Instituto de Agrobiotecnología, Universidad Pública de Navarra-Consejo Superior de Investigaciones CientíficasPamplona, Spain; ^5^UMR 8618 Institut de Biologie des Plantes, Centre National de la Recherche Scientifique, Université Paris-SudOrsay, France

**Keywords:** antioxidant mechanisms, oxidative stress, photosynthesis, redox homeostasis, thioredoxin, tobacco

## Abstract

Plants display a remarkable diversity of thioredoxins (Trxs), reductases controlling the thiol redox status of proteins. The physiological function of many of them remains elusive, particularly for plastidial Trxs f and m, which are presumed based on biochemical data to regulate photosynthetic reactions and carbon metabolism. Recent reports revealed that Trxs f and m participate *in vivo* in the control of starch metabolism and cyclic photosynthetic electron transfer around photosystem I, respectively. To further delineate their *in planta* function, we compared the photosynthetic characteristics, the level and/or activity of various Trx targets and the responses to oxidative stress in transplastomic tobacco plants overexpressing either Trx f or Trx m. We found that plants overexpressing Trx m specifically exhibit altered growth, reduced chlorophyll content, impaired photosynthetic linear electron transfer and decreased pools of glutathione and ascorbate. In both transplastomic lines, activities of two enzymes involved in carbon metabolism, NADP-malate dehydrogenase and NADP-glyceraldehyde-3-phosphate dehydrogenase are markedly and similarly altered. In contrast, plants overexpressing Trx m specifically display increased capacity for methionine sulfoxide reductases, enzymes repairing damaged proteins by regenerating methionine from oxidized methionine. Finally, we also observed that transplastomic plants exhibit distinct responses when exposed to oxidative stress conditions generated by methyl viologen or exposure to high light combined with low temperature, the plants overexpressing Trx m being notably more tolerant than Wt and those overexpressing Trx f. Altogether, these data indicate that Trxs f and m fulfill distinct physiological functions. They prompt us to propose that the m type is involved in key processes linking photosynthetic activity, redox homeostasis and antioxidant mechanisms in the chloroplast.

## INTRODUCTION

Thioredoxins (Trxs) are ubiquitous and evolutionarily conserved enzymes of *ca*. 12 kDa catalyzing the reduction of disulfide bonds through a redox-active dithiol CxxC motif (Arnér and Holmgren, 2000). Trxs, discovered 50 years ago in bacteria, cover functions as redox carriers in numerous physiological processes such as DNA synthesis, sulfur assimilation or regulation of transcription factors (Arnér and Holmgren, 2000). In plants, two plastidial Trx types, named Trx f and Trx m, were primarily identified as light-dependent regulators of enzymes related to photosynthetic processes and carbon metabolism (Jacquot et al., 1978; Wolosiuk et al., 1979). Their denomination was based on *in vitro* ability to activate by reduction fructose-1,6-bisphosphatase (FBPase) and NADP-dependent malate dehydrogenase (NADP-MDH), respectively. These Trxs are reduced by the ferredoxin/thioredoxin system (Wolosiuk and Buchanan, 1977). A third plant Trx type located in cytosol was identified later (Wolosiuk et al., 1979). This type named Trx h is reduced by cytosolic NADPH thioredoxin reductase (Jacquot et al., 1994) and participates in various processes such as mobilization of seed reserves (Kobrehel et al., 1992) and responses to oxidative stress (Verdoucq et al., 1999).

Whereas most organisms possess a low number (two or three) of Trxs achieving multiple functions, plants display a remarkable diversity of these reductases. A survey of genomic and EST sequences from *Arabidopsis* and other species revealed the presence of almost 50 genes encoding Trx or Trx-like proteins in higher plants (Meyer et al., 2005). On the basis of gene and peptide sequences, other Trx types, o, x, y, and z, have been defined and in *Arabidopsis*, Trxs f, h, m and y include 2, 9, 4, and 2 isoforms, respectively (Meyer et al., 2005; Lemaire et al., 2007). This outstanding diversity raised the question of functional specialization or redundancy. Genetic studies revealed that several plant Trxs possess specific and unique physiological functions in development, metabolism and stress responses. For instance, *Arabidopsis* mutants deficient in Trx h9 display impaired root and leaf development. This cytosolic Trx is associated with plasma membrane and presumed to participate in cell-to-cell communication processes (Meng et al., 2010). In *Arabidopsis* plants lacking plastidial Trx z, chloroplast biogenesis is inhibited revealing a critical role of this Trx (Arsova et al., 2010). Trx z has been proposed to regulate transcription via a redox control of plastid-encoded plastid RNA polymerase. In other respects, Trx h5 is required for the response to victorin, a fungal toxin inducing programmed cell death in sensitive plants (Sweat and Wolpert, 2007).

Other Trxs participate in plant responses to the oxidative stress conditions resulting from environmental constraints (Vieira Dos Santos and Rey, 2006), mainly due to their ability to provide reducing power to peroxiredoxins (Prxs) and methionine sulfoxide reductases (MSRs), enzymes reducing organic peroxides and repairing oxidized proteins, respectively. Thus, CDSP32 (chloroplastic drought-induced stress protein of 32 kDa), a double module Trx initially isolated in potato plants subjected to water deficit (Rey et al., 1998) supplies Prxs and MSRs with electrons (Broin et al., 2002; Rey et al., 2005; Tarrago et al., 2010). Another plastidial Trx-like protein, NADPH thioredoxin reductase C (NTRC), uses NADPH to reduce 2-Cys Prx and has been proposed as a protection system against oxidative damage (Pérez-Ruiz et al., 2006). Trxs x and y are also presumed to participate in responses to oxidative stress based on their ability to reduce Prxs and MSRs *in vitro* (Collin et al., 2003, 2004; Navrot et al., 2006; Vieira Dos Santos et al., 2007), but evidence for such a function *in planta* is still scarce. Very recently, we showed that Trx y2 maintains growth under high light and long day in *Arabidopsis*, likely through electron supply to plastidial MSRs (Laugier et al., 2013). Note that the other Trx y isoform, y1, which is specifically expressed in non-photosynthetic organs (Collin et al., 2004) could also fulfill a protective function in seeds, in which MSRs likely play a key role in preserving longevity (Châtelain et al., 2013).

Although Trxs f and m have been the first Trxs discovered in plants, the knowledge concerning their physiological functions is only emerging. Based on biochemical studies, Trxs f and m are presumed to regulate photosynthesis and carbon metabolism although Trx f seems more efficient than Trx m to redox regulate most enzymes involved in these processes. Trx f specifically activates glyceraldehyde-3-phosphate dehydrogenase (B-containing GAPDH isoforms) and FBPase, and controls the activity of other redox-sensitive enzymes like NADP-MDH and glucose-6-phosphate dehydrogenase (G6PDH; Collin et al., 2003; Lemaire et al., 2007; Marri et al., 2009; Née et al., 2009). Similarly, Trx m reduces enzymes involved in carbon metabolism and catabolism such as NADP-MDH and G6PDH, but also regenerates the activity of enzymes involved in antioxidant mechanisms like Prxs and MSRs (Collin et al., 2003; Vieira Dos Santos et al., 2007). Among Trx m isoforms, Trx m3 displays highly distinct properties, since it cannot reduce known Trx targets (Collin et al., 2003; Vieira Dos Santos et al., 2007). This isoform, expressed in non-green plastids of meristems and organ primordia, could be involved in redox regulation of symplastic permeability (Benitez-Alfonso et al., 2009). Using RNA-interference, Chi et al. (2008) showed that rice plants knockdown for Trx m expression display abnormal chloroplast development and impaired growth. In contrast, no obvious phenotype was observed in *Arabidopsis* plants lacking either Trx m1 or Trx m4 (Laugier et al., 2013). But, most interestingly, Trx m4-deficient mutant plants specifically display strongly increased cyclic photosynthetic electron transfer around PSI (Courteille et al., 2013). Only very recent papers brought information regarding the physiological function of Trx f. In pea plants displaying a dramatically reduced Trx f transcript level due to silencing, no phenotype was noticed (Luo et al., 2012). Similarly, no change was found in growth and photosynthesis in *Arabidopsis* knockout lines for Trx f1, but reduced light-activation of ADP-glucose pyrophosphorylase (AGPase) in leaves accompanied by a decrease in starch accumulation was observed in these mutants (Thormählen et al., 2013). Consistently, we observed that transplastomic tobacco plants overexpressing Trx f show a strong increase in starch content (Sanz-Barrio et al., 2013).

The data gained in various species indicate that Trx f is involved in the regulation of starch metabolism, whereas the role of Trx m seems more complex. In this work, we compare the phenotypes of tobacco plants overexpressing either Trx f or Trx m with regards to growth, photosynthetic metabolism, activation and content of Trx targets and response to oxidative stress. We show that overexpression of Trx m leads to delayed growth, reduced pigment content and impaired photosynthetic activity. Further, we found a differential behavior of plants overexpressing Trx f or Trx m exposed to oxidative stress conditions, revealing that Trx m very likely displays, compared to Trx f, a broader range of physiological functions.

## MATERIALS AND METHODS

### PLANT MATERIAL AND STRESS TREATMENTS

*Nicotiana tabacum *L.**plants, cv Petit Havana (Wt and transplastomic lines), were sown and grown on compost in phytotron under a 12-h photoperiod (300 μmol photons m^-2^ s^-1^) and a 25°C/19°C (day/night) temperature regime for standard conditions. Transplastomic plants overexpressing either Trx f or Trx m were generated and characterized as reported in Sanz-Barrio et al. (2013). Photosynthetic and biochemical analyses were carried out on young well-expanded leaves from 35- to 40-day old plants.

Photooxidative treatment was carried out by exposing 30-day-old tobacco plants grown under standard conditions to high light intensity (950 μmol photons m^-2^ s^-1^) and low temperature (8°C) for 5 to 8 days under a 12-h photoperiod. Methyl viologen (MV) treatment was performed both on whole plants and leaf disks. For whole plant experiments, 40-day-old tobacco plants were sprayed with 30 μM MV in 0.05% (v/v) Tween 20 and placed in phytotron under 200 μmol photons m^-2^ s^-1^ and a 16-h light (28°C)/8-h dark (25°C) photoperiod. Leaf damage caused by MV was photographed 2 days after treatment. For leaf disk experiments, 15 disks (12 mm diameter) were punched from young fully expanded leaves from 40-day-old plants, floated topside up on 15 mL of water or 1 μM MV, and illuminated at 600 μmol photons m^-2^ s^-1^ and 28°C during 14 h. Electrolyte content in solution was measured after treatment using a HI9813-5 conductivity meter (Hanna Instruments, Woonsocket, RI, USA). Total electrolyte content was determined in the same way after autoclaving samples. Results were expressed as the percentage of total electrolytes released after treatment.

### PHOTOSYNTHETIC MEASUREMENTS

Chlorophyll fluorescence parameters were measured using a PAM-2000 modulated fluorometer (Waltz Effeltrich, Germany) as previously described (Havaux et al., 2000). A saturating pulse of white light was applied on leaf and measurements were recorded during actinic light illumination (from 25 to 2,500 μmol photons m^-2^ s^-1^). The PSII photochemical efficiency (Φ_PSII_) during actinic illumination was estimated by calculating Δ*F*/*F*_m__′_, where Δ*F* is the steady-state chlorophyll fluorescence level and *F*_m__′_ is the maximal level. NPQ (non-photochemical quenching) reflects the dissipation of absorbed light energy from PSII as heat. NPQ was calculated as (*F*_m_/*F*_m__′_)-1 where *F*_m_ is the maximal fluorescence level in the dark.

### ANALYSIS OF CHLOROPHYLL CONTENT

One-cm diameter leaf disks were collected from fully expanded mature leaves and immediately frozen in liquid nitrogen and stored at -80°C until use. Leaf disks were crushed in 1 mL 80% acetone. After storing overnight in the dark at 4°C and centrifugation (14,000 *g*, 10 min), the content in chlorophylls *a* and *b* was measured spectrophotometrically and calculated according to Lichtenthaler (1987).

### GLUTATHIONE CONTENT

Three tobacco leaf disks of 1 cm diameter (about 100 mg) were ground to a fine powder in liquid nitrogen and extracted in 1 mL 6.3 mM diethylene triamine-pentaacetic acid (DTPA), 40 μM *N*-acetyl-L-cysteine and 0.15% trifluoroacetic acid (TFA). After centrifugation (15,000 *g*, 10 min), the supernatant containing non-protein thiols was filtered on 0.2 mm nylon membrane. 125 μl were added to 225 μL buffer A (6.3 mM DTPA, 0.2 M 4-(2-hydroxyethyl)-piperazine-1-propane-sulfonic acid, pH 8.2) or buffer B (buffer A + 0.5 mM Tris(2-carboxy-ethyl)phosphine hydrochloride, TCEP). For measurements of GSH content, samples in buffer A were immediately alkylated with monobromobimane in acetonitrile at a final concentration of 500 μM and stabilized by adding 150 μL cold 1 M methane sulfonic acid following 20 min incubation in the dark. For measurements of total glutathione (GSH), samples in buffer B were alkylated for 45 min at room temperature. Reactions were stopped by adding 150 μL of cold 1 M methane sulfonic acid. 20 μl were analyzed by HPLC and measurements of the fluorescence of bimane derivates were carried out as in Collin et al. (2008). Quantification of GSH amount was based on peak area and calibration was performed using GSH (Sigma). The concentration of oxidized glutathione (GSSG) was calculated as the difference between total GSH and reduced GSH values.

### ASCORBIC ACID CONTENT

Ascorbate (AsA) was analyzed by HPLC as described by Havaux et al. (2005). Three leaf disks of 1 cm in diameter were ground in 750 μL 0.1 M metaphosphoric acid. Samples were filtered on nylon 0.2 μM membrane (Spin-X Costar). 6 μL were immediately injected for assaying reduced ascorbate. Total ascorbate was measured in the same volume following reduction of dehydroascorbic acid into ascorbic acid using 10 mM TCEP for 2 h in the dark at 25°C. AsA was detected at 245 nm in sulfuric acid-acidified water (pH 2.5) at a retention time of 1 min under a 0.65 mL min^-1^ flow. Quantification of AsA amount was based on peak area and calibration was performed using AsA from Sigma.

### PROTEIN EXTRACTION, SDS-PAGE AND WESTERN ANALYSIS

Leaf pieces were blended in liquid nitrogen, and the powder was used to prepare soluble proteins as described in Rey et al. (2005). The protein content was determined using the BC Assay Reagent (Interchim, Montluçon, France). Proteins were separated using SDS-PAGE and Coomassie Brilliant Blue staining of gels was carried out to check quality of protein extracts. For immunoblot analysis following electrophoresis, proteins were electro-transferred onto nitrocellulose membrane (Pall Corporation, Ann Arbor, MI, USA) and Red Ponceau staining was achieved to ensure equal loading in all lanes. Western analysis was carried out using primary antibodies raised in rabbit against NtTrx m or NtTrx f (1:5,000; Sanz-Barrio et al., 2011), AtMSRB1 and AtMSRB2 (1:1,000; Laugier et al., 2010), poplar PrxQ (1:2,000; Rouhier et al., 2004) and catalase (1:1,000; Agrisera, Vännas, Sweden). Western analysis of 2-Cys Prx abundance and redox status was performed as in Rey et al. (2007). Bound antibodies were detected using either an anti-rabbit IgG alkaline phosphatase conjugate (Sigma-Aldrich) or a goat anti-rabbit “Alexa Fluor^®^ 680” IgG from Invitrogen diluted 1:10,000. When using the latter, antibodies were revealed at 680 nm using the “Odyssey Infrared Imager” from Licor.

### NADP-MDH AND NADP-GAPDH ACTIVITY ASSAYS

NADP-dependent malate and glyceraldehyde-3-phosphate dehydrogenases activities in tobacco leaf crude extracts were assayed spectrophotometrically as previously described in Keryer et al. (2004) and Marri et al. (2009), respectively. Extractable enzymatic activities were measured on aliquots of freshly prepared extracts and maximal activities were measured after pre-treatment (reductive activation) of extracts with 25 mM DTT for 20 min at room temperature.

### MSR ACTIVITY ASSAY

Maximal MSR activity in tobacco leaf extracts was assayed by monitoring the reduction of the synthetic substrate, dabsyl-MetO, in the presence of DTE (Vieira Dos Santos et al., 2005; Laugier et al., 2010). After blending leaves and suspension in extraction buffer, the content in soluble proteins was determined as above. The reaction mixture contained 15 mM HEPES pH 8, 10 mM MgCl_2_, 30 mM KCl, 20 mM DTE, 0.25 mM dabsyl-MetO and 30 or 300 μg soluble proteins. After incubation for 3 h at 37°C, stopping using an ethanol:acetate buffer (50:50) and centrifugation, a supernatant aliquot was loaded on a C18 reverse phase 3.5 μm, 3 mm × 50 mm column SunFire^TM^ (Waters, Milford, MA, USA) to separate dabsyl-MetO and dabsyl-Met.

## RESULTS

### GROWTH CHARACTERISTICS OF TOBACCO PLANTS OVEREXPRESSING Trx f OR Trx m

**FIGURE 1:**
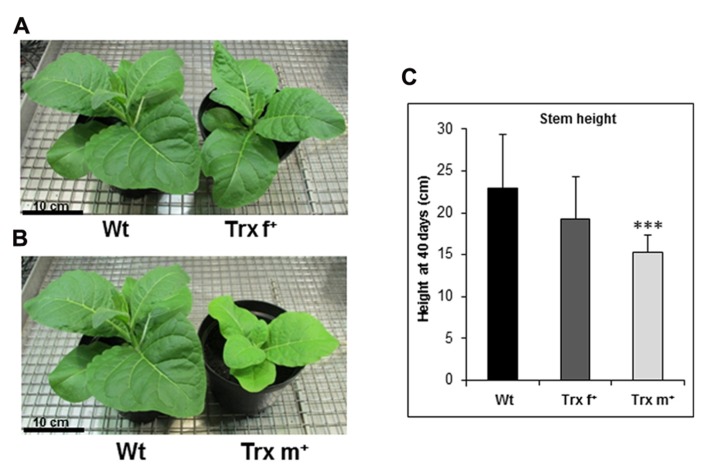
**Growth of transplastomic tobacco plants overexpressing Trx f or Trx m.** **(A)** Wt and Trx f^+^ plants grown in phytotron for 35 days in standard conditions (300 μmol photons m^-2^ s^-1^, 12-h photoperiod and 25°C/19°C, day/night). **(B)** Wt and Trx m^+^ plants grown in phytotron for 35 days under standard conditions. The same Wt plant is shown in panels **(A)** and **(B)**. **(C)** Stem height of Wt and transplastomic plants grown for 40 days in standard conditions. Mean stem height values ±SD were gained from 15 plants per genotype. Wt, wild-type; Trx f^+^ and Trx m^+^, plants overexpressing Trx f or Trx m, respectively. ***Significantly different from Wt with *p* < 0.001 (*t*-test).

The transplastomic tobacco plants studied in this work were generated as described previously (Sanz-Barrio et al., 2013) by inserting tobacco Trx f or Trx m sequences (GenBank Acc. N° HQ338526 and HQ338525, respectively) without predicted transit peptides in the chloroplast genome under the control of the *psbA* regulatory sequence. Compared to Wt, the tobacco transformant lines termed Trx f^+^ and Trx m^+^ were shown to contain strongly increased Trx protein levels (at least 20 times more for Trx f^+^ and 15 times for Trx m^+^; Sanz-Barrio et al., 2013 and data not shown). The first phenotype analysis revealed that compared to Wt and Trx f^+^ plants, Trx m^+^ plants display some delay (2–3 days) in germination in *in vitro* conditions, a pale-green phenotype and a delay of few days in flowering time when grown in greenhouse conditions (Sanz-Barrio et al., 2013). In the present work, we further investigated the growth parameters of the transplastomic lines grown on compost in phytotron conditions. We noticed that plants overexpressing Trx m do not exhibit any delay in germination in these conditions, but show a slower growth compared to Wt and those overexpressing Trx f (**Figures 1A–C**). Thus at 40 days, the height stem of Trx m^+^ plants (15.3 ± 2.1 cm) is significantly lower than that of Wt plants (22.9 ± 6.5 cm), whereas the growth of Trx f^+^ plants is slightly altered, their height stem being 19.3 ± 5 cm (**Figure 1C**). The slower growth of Trx m^+^ plants is associated with some delay (*ca.* 3 days) in flowering, but the plant size at this developmental stage is very similar to that of Wt and Trx f^+^ plants (data not shown), as previously observed (Sanz-Barrio et al., 2013). We determined the leaf specific weight and did not notice any difference for the three lines (**Table 1**). Regarding the dry matter percentage, we observed a slightly, but significantly higher ratio in Trx f^+^ plants (**Table 1**). This characteristic could originate from the much higher starch content measured in this line (Sanz-Barrio et al., 2013).

**Table 1:** Leaf specific weight, dry matter percentage and chlorophyll content in 40-day old Wt and transplastomic tobacco plants.

Genotype	Wt	Trx f^+^	Trx m^+^
Leaf specific weight (mg FW cm^-^^2^)	21.4 ± 0.9	20.8 ± 1.6	21.0 ± 1.7
% Dry matter	10.3 ± 0.8	11.4 ± 1.1*	10.2 ± 1.3
Chl (μg cm^-^^2^)	44.6 ± 1.4	41.1 ± 1.0**	30.9 ± 1.3***
Chl *a* (μg cm^-^^2^)	31.6 ± 1.7	28.9 ± 1.0*	23.4 ± 1.0***
Chl *b* (μg cm^-2^)	13.0 ± 0.6	12.2 ± 1.1	7.6 ± 0.3***
Chl *a*/Chl *b*	2.4 ± 0.2	2.4 ± 0.2	3.1 ± 0.1***

Chlorophyll content was measured in young well-expanded leaves of 40-day old Wt and transplastomic tobacco plants. Chlorophyll data are mean values ± SD from five independent measurements per plant and genotype. Leaf specific weight and dry matter data are mean values ± SD from eight independent measurements per plant and genotype. Each measurement was carried out using three 1-cm leaf disks. Wt, wild-type; Trx f^+^ and Trx m^+^, plants overexpressing Trx f or Trx m, respectively. Values significantly different from Wt values with *p > 0.05, **p > 0.01, and ***p > 0.001 (*t*-test).

We then measured chlorophyll content in fully expanded leaves (**Table 1**) and observed that Trx f^+^ plants display a pigment content slightly, but significantly, lower than that of Wt (41.1 ± 1.0 against 44.6 ± 1.4 μg cm^-2^). This difference has not been observed when plants were grown in greenhouse under longer photoperiod and higher temperature (Sanz-Barrio et al., 2013). Thus, we presume that this phenotype feature originates from the different environmental conditions used in the present study. Consistent with visual observations, a chlorophyll content reduced by more than 30% (30.9 ± 1.3 μg cm^-2^) compared to Wt was recorded in Trx m^+^ plants. Note that when plants were grown at higher temperature, the pigment content was reduced in a less pronounced manner (-25%; Sanz-Barrio et al., 2013). The chlorophyll *a*/chlorophyll *b* ratio was similar (2.4) in both Wt and Trx f^+^ plants, but higher (3.1) in Trx m^+^ plants (**Table 1**), due to a chlorophyll *b* content reduced to a larger extent (-42% compared to Wt) than that of chlorophyll *a* (-26%; **Table 1**).

### PHOTOSYNTHETIC PROPERTIES OF TOBACCO PLANTS OVEREXPRESSING Trx f OR Trx m

**FIGURE 2:**
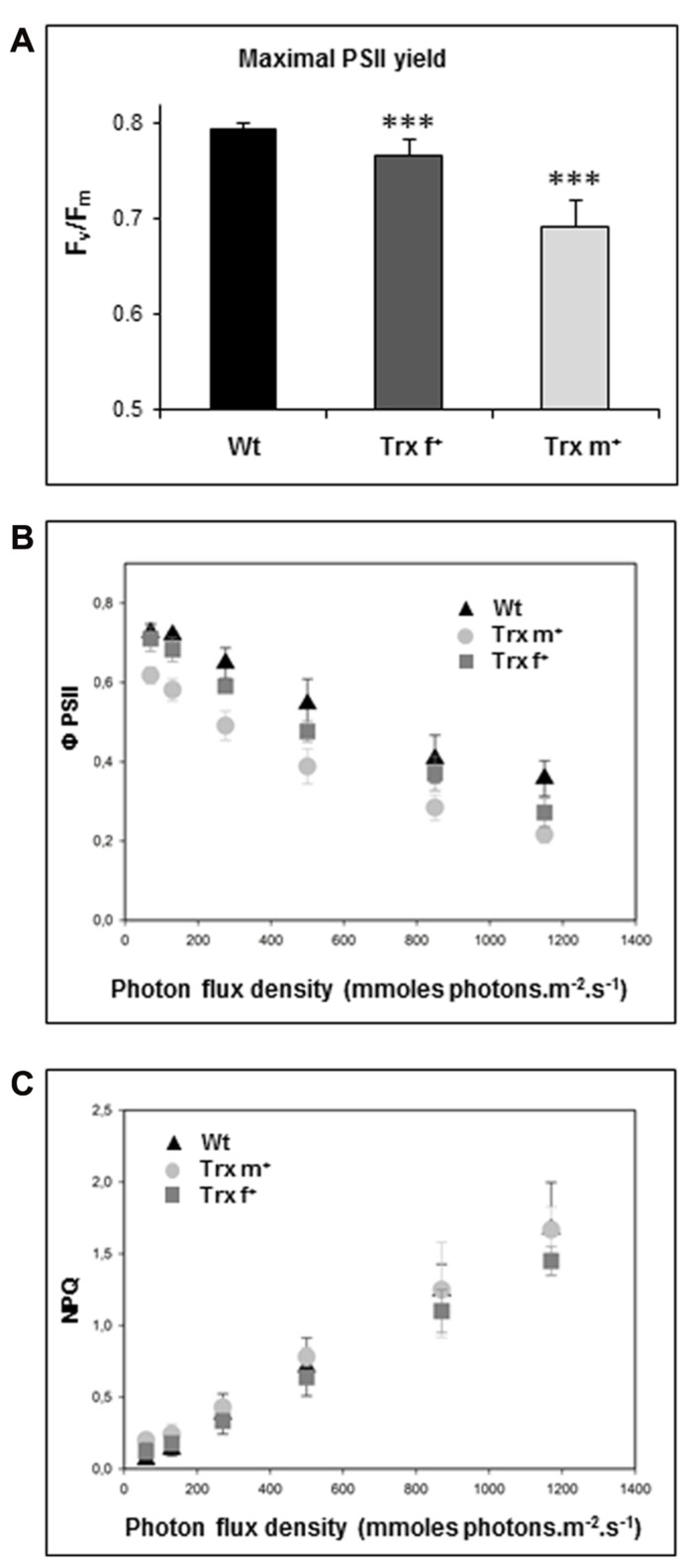
**Photosynthetic fluorescence parameters of transplastomic tobacco plants overexpressing Trx f or Trx m.** **(A)** Maximal photosystem II photochemical efficiency in tobacco plants (*F*_v_/*F*_m_). **(B)** Effective photosystem II yield (ΦPSII). **(C)** Non-photochemical quenching (NPQ). Light response curves are shown for ΦPSII **(B)** and NPQ **(C)**. Measurements were achieved in leaves from 35-day-old Wt and transplastomic tobacco plants grown in standard conditions. Data are mean values ± SD from at least five independent measurements per genotype. Wt, wild-type; Trx f^+^ and Trx m^+^, plants overexpressing Trx f or Trx m, respectively. ***Significantly different from Wt with *p* < 0.001 (*t*-test).

We then investigated the photosynthetic properties of transplastomic tobacco plants and first measured maximal PSII photochemical efficiency (**Figure 2A**) by recording the chlorophyll fluorescence parameter, *F*_v_/*F*_m_, which reflects the PSII capacity to reduce the primary *Q*_A_ electron acceptor. We observed a typical value close to 0.8 in Wt plants (0.793 ± 0.007). A slightly altered *F*_v_/*F*_m_ value (0.766 ± 0.017) was recorded in the Trx f^+^ line, whereas this fluorescence parameter was noticeably reduced in plants overexpressing Trx m (0.692 ± 0.028). These data reveal impairment in PSII functioning in plants accumulating Trx m. Photosynthetic electron transport activity was also estimated by measuring Φ_PSII_, a chlorophyll fluorescence parameter indicative of the efficiency of PSII photochemistry as a function of light intensity. While only slightly decreased in Trx f^+^ plants, PSII photochemical performance appeared more impaired in plants overexpressing Trx m (**Figure 2B**). Thermal dissipation of absorbed light energy, expressed by the NPQ of chlorophyll fluorescence coefficient, was also recorded in the different lines (**Figure 2C**). No noticeable difference was detected between Wt and Trx^+^ transplastomic lines since they displayed a similar ability to recover photosynthesis and PSII photochemical efficiency following light irradiation.

### ACTIVITY OF Trx TARGETS INVOLVED IN CARBON METABOLISM

**FIGURE 3:**
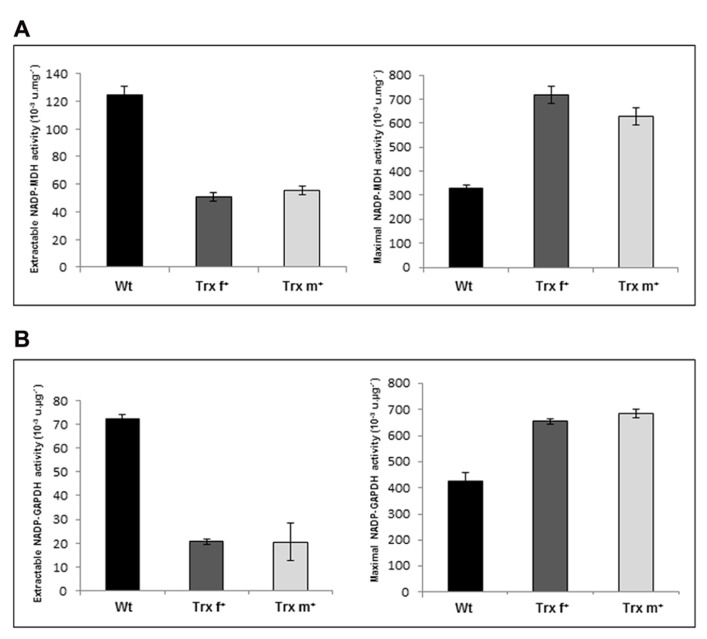
**NADP-dependent malate and glyceraldehyde-3-phosphate dehydrogenases enzymatic activities in leaf extracts from transplastomic tobacco plants overexpressing Trx f or Trx m.** **(A)** Extractable and maximal NADP-MDH activities. **(B)** Extractable and maximal NADP-GAPDH activities. Maximal activities (capacities) were measured after a reducing treatment of protein leaf extracts (25 mM DTT, 20 min at room temperature). Data are mean values ± SD from at least four independent measurements per genotype. Wt, wild-type; Trx f^+^ and Trx m^+^, plants overexpressing Trx f or Trx m, respectively.

To get evidence that overexpression of plastidial Trxs has a functional impact *in planta* on Trx target proteins, we first measured in leaves of transplastomic lines the activities of two well-known Trx-regulated enzymes, NADP-dependent MDH and GAPDH (Lemaire et al., 2007). Both Trx f^+^ and Trx m^+^ plants show similar marked changes in enzymatic activities. Extractable leaf activities were very strongly lowered to *ca.* 40 and 30% of the Wt value for MDH and GAPDH, respectively (**Figures 3A, B**, left panels). Conversely, leaf MDH and GAPDH capacities corresponding to the maximal activities measured in extracts chemically reduced with DTT, which allows Trx reduction but not direct Trx-target activation, were substantially increased in both transplastomic lines (**Figures 3A,B**, right panels). These findings show that overexpressed Trxs modulate the activity of known targets *in planta*. Further, they indicate that both Trx f and m, when they are in large excess, regulate NADP-dependent MDH and GAPDH enzymes in a similar manner.

### Prx AND MSR ABUNDANCE

**FIGURE 4:**
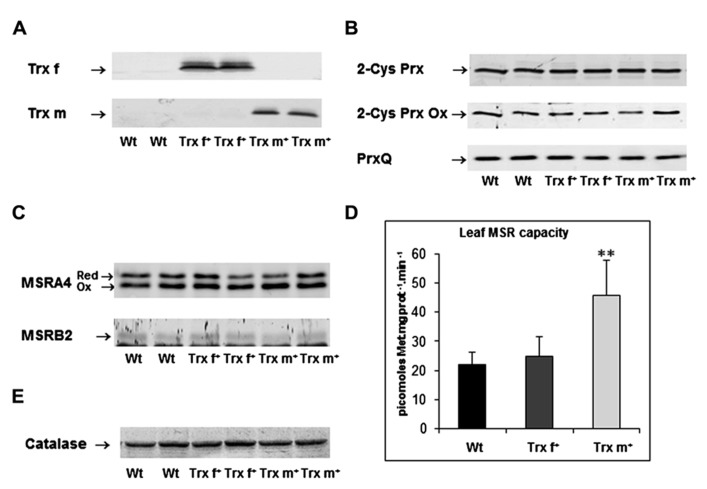
**Abundance of proteins involved in antioxidant mechanisms and leaf peptide methionine sulfoxide reductase enzymatic capacity in leaf extracts from transplastomic tobacco plants overexpressing Trx f or Trx m.** **(A)** Western analysis of Trx f and Trx m abundance. **(B)** Western analysis of the abundance and redox status of plastidial 2-Cys peroxiredoxin (2-Cys Prx) and of the abundance of peroxiredoxin Q (PrxQ). **(C)** Western analysis of the abundance of plastidial methionine sulfoxide reductases A4 and B2, MSRA4 and MSRB2, respectively. **(D)** Peptide methionine sulfoxide reductase enzymatic capacity measured using HPLC in crude leaf extracts using dabsyl-MetO, a synthetic substrate mimicking peptide-bound MetO. Data are mean values ± SD from at least five independent measurements per genotype. **Significantly different from Wt with *p* < 0.01 (*t*-test). **(E)** Western analysis of catalase abundance. Similar Western data were obtained using leaf extracts from two independent plants originating from at least two distinct growth experiments. Wt, wild-type; Trx f^+^ and Trx m^+^, plants overexpressing Trx f or Trx m, respectively.

Peroxiredoxins are ubiquitous thiol-based peroxidases detoxifying hydrogen and organic peroxides. In plants, several Trx types such as NTRC, Trx x, CDSP32 and Trx y supply with electrons the main Prx plastidial type, 2-Cys Prx, and another isoform, PrxQ (Dietz, 2011). We thus investigated the abundance of these Prxs in tobacco plants by performing Western analysis. First, we confirmed the high abundance of Trxs f and m in transplastomic lines (**Figure 4A**). The antibodies raised against *Arabidopsis* 2-Cys Prx and poplar PrxQ homologues specifically revealed bands with the expected molecular masses in tobacco extracts (**Figure 4B**). No change was detected in the amount of the two plastidial Prxs in Trx^+^ plants (**Figure 4B**). We also used antibodies raised against overoxidized 2-Cys Prx, an inactivated form possibly involved in signaling processes related to redox homeostasis (Rey et al., 2007). Whereas there was no change in 2-Cys Prx abundance in plants overexpressing Trx f or Trx m compared to Wt, we observed a more variable amount of overoxidized 2-Cys Prx in the same leaf extracts. We noticed that such a variation occurred within the same genetic background (**Figure 4B**). A quantitative analysis, performed on data gained from six independent plants per line (data not shown), lead us to conclude that there was no significant variation in the 2-Cys Prx redox status in transplastomic tobacco plants.

Methionine sulfoxide reductase enzymes, which repair oxidized methionine in proteins, are also well-known targets of Trxs involved in plant responses to environmental constraints (Laugier et al., 2010). The abundance of plastidial MSRs was investigated in tobacco plants using sera raised against *Arabidopsis* MSRB1, MSRB2 and poplar MSRA4. MSRA4 antiserum recognized two bands at *ca.* 25 kDa in tobacco extracts (**Figure 4C**), corresponding to the reduced and oxidized forms of plastidial MSRA as observed in other species (Vieira Dos Santos et al., 2005; Bouchenak et al., 2012; Marok et al., 2013). No substantial change was observed regarding the abundance of MSRA4 forms in Trx-overexpressing plants compared to Wt. For MSRB1, no unambiguous signal could be detected in tobacco extracts due to poor cross reactivity of the serum generated against *Arabidopsis* homologue (data not shown). Like in *Arabidopsis* extracts, a faint band at *ca.* 15 kDa was revealed for MSRB2, next to a lower non-specific and intense band (Laugier et al., 2010). Again, overexpression of either Trx f or Trx m was not found to result in any substantial change in the amount of this plastidial MSR isoform. Altogether, Western data reveal that the levels of some major Trx targets involved in antioxidant mechanisms are not modified when Trxs f and m are overproduced.

### LEAF MSR CAPACITY

We then measured the MSR enzymatic capacity in leaf extracts from tobacco plants using dabsyl-MetO, a substrate mimicking peptide-bound MetO (**Figure 4D**). In Wt, a maximal activity in the range of 22 pmol Met. mg prot^-1^ min^-1^ was measured in the presence of reductant. Note that this value is noticeably lower than that measured in *Arabidopsis*, *ca. *50 pmol Met. mg prot^-1^ min^-1^ (Laugier et al., 2010), but higher than that recorded in barley, *ca.* 10 pmol Met. mg prot^-1^ min^-1^ (Marok et al., 2013). In Trx f^+^ plants, a rather similar value was found, *ca.* 25 pmol Met. mg prot^-1^ min^-1^. In sharp contrast, a twice higher value (46 pmol Met. mg prot^-1^ min^-1^) was measured in plants overexpressing Trx m. These data, showing a more elevated MSR capacity in Trx m^+^ plant extracts, reveal that *in planta* Trx m, but not Trx f, very likely regenerates and sustains the activity of plastidial MSRs.

### CATALASE ABUNDANCE

The previous data revealed that Trx m likely provides reducing power to MSRs. MSRs are enzymes repairing oxidized proteins, but could also play a more general antioxidant function since their action results in ROS scavenging at the expense of NADPH (Moskovitz et al., 1997). To further analyze a putative function of plastidial Trxs in the control of redox homeostasis in plant cells, we analyzed leaf catalase abundance using Western blot analysis. Catalase is one major enzymatic system responsible for H_2_O_2_ scavenging in plant cells. We did not notice any substantial difference in catalase amount in plants overexpressing either Trx f or Trx m compared to Wt (**Figure 4E**), likely indicating no important change in the catalase-based capacity for detoxifying H_2_O_2_ in these lines.

### CONTENT AND REDOX STATUS OF GLUTATHIONE AND ASCORBATE

**FIGURE 5:**
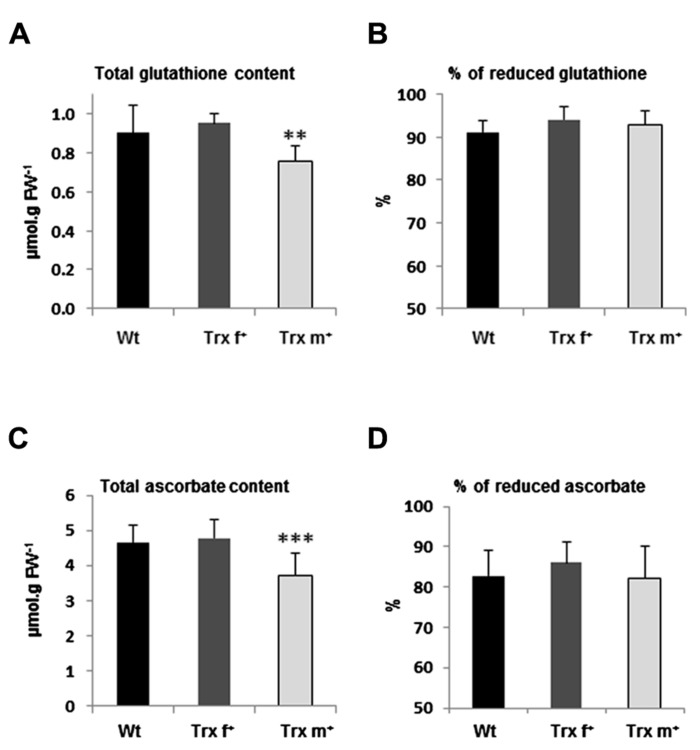
**Glutathione and ascorbate content in transplastomic tobacco plants overexpressing Trx f or Trx m.** Total content **(A,** **C)** and redox status **(B,** **D)** of glutathione **(A,** **B)** and ascorbate **(C,** **D)** were determined in leaves of tobacco plants grown in standard conditions for 40 days. Measurements were performed using HPLC as described in Section “Materials and Methods” using three 1-cm leaf disks from young well-expanded leaves. Data are mean values ± SD from at least 10 independent measurements per genotype. Wt, wild-type; Trx f^+^ and Trx m^+^, plants overexpressing Trx f or Trx m, respectively. Values significantly different from Wt values with **p < 0.01 and ***p < 0.001 (*t*-test).

We then investigated whether non-enzymatic antioxidant systems could be altered in transplastomic tobacco lines and measured the leaf content in GSH and ascorbate, which are abundant soluble antioxidants fulfilling key roles in redox homeostasis (Noctor and Foyer, 1998). In young well-expanded leaves of 40-day-old Wt tobacco plants, a GSH content of *ca.* 0.90 μmol g FW^-1^ was measured, the proportion of reduced form being 91% (**Figures 5A,B**). In Trx f^+^ plants, the content and percentage values were very similar: 0.95 μmol g FW^-1^ and 94%, respectively. Interestingly, in plants overexpressing Trx m, whereas the proportion of reduced GSH was not modified (93%), the content, 0.75 μmol g FW^-1^, was significantly lower (*ca.* -20%) than in Wt and Trx f^+^ plants (**Figures 5A,B**). Regarding ascorbate, both Wt and Trx f^+^ plants display very similar total contents (0.82 and 0.85 μnmol g FW^-1^, respectively) and proportions of reduced form (83 and 86%, respectively) in young well-expanded leaves (**Figures 5C,D**). In contrast, the total ascorbate content, 0.65 μnmol g FW^-1^, was significantly lower in Trx m^+^ plants, but with an unchanged redox status (82%) compared to the two other lines (**Figures 5C,D**). Taken together, these data reveal that GSH and ascorbate pools are not altered when Trx f is overexpressed, but significantly and similarly modified in Trx m^+^ plants. Indeed, these plants exhibit a decrease of *ca.* 20% in the total content of ascorbate and GSH without any noticeable change in redox status.

### TOLERANCE TO PHOTOOXIDATIVE TREATMENT OF TOBACCO PLANTS OVEREXPRESSING Trx f OR Trx m

**FIGURE 6:**
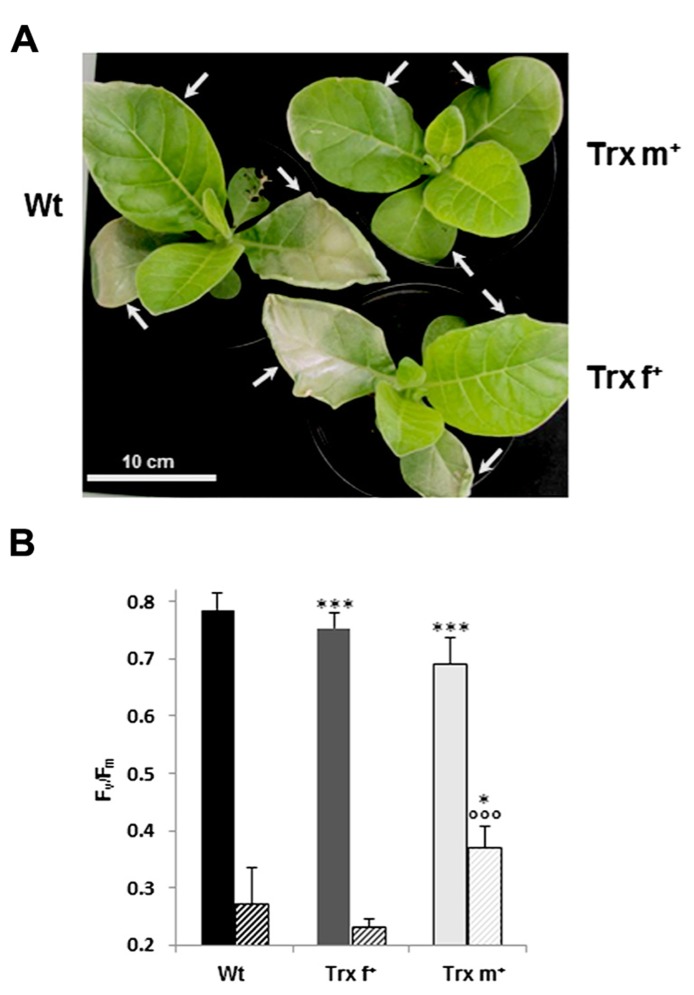
**Tolerance to photooxidative treatment of transplastomic tobacco plants overexpressing Trx f or Trx m.** Photooxidative treatment was carried out by exposing 30-old tobacco plants grown in standard conditions to high light intensity (950 μmol photons m^-2^ s^-1^) and low temperature (8°C) under a 12-h photoperiod in a controlled growth chamber. **(A)** Photograph showing representative plants subjected to the treatment for 5 days. Three independent experiments were carried out with three plants per genotype in each experiment. **(B)** Maximal photosystem II photochemical efficiency in tobacco plants. *F*_v_/*F*_m_ values were measured in plants grown in control conditions for 32 days (full bars) and in plants further subjected to photooxidative conditions for 5 days (dashed bars). Data are mean values ± SD of the average values from eight measurements performed on five plants per genotype. Measurements were performed on representative areas from three leaves (indicated by arrows in panel **A**) per plant. Wt, wild-type; Trx f^+^ and Trx m^+^, plants overexpressing Trx f or Trx m, respectively. Values significantly different from Wt values with *p < 0.05 and ***p < 0.001 (*t*-test); °°°Value significantly different from the Trx f^+^ value with *p* < 0.001 (*t*-test).

Oxidative stress tolerance of transplastomic tobacco plants was monitored by exposing them to a treatment combining high light and low temperature, conditions known to generate loss of photosynthetic membranes within chloroplasts due to photooxidative damage. Thirty-day old plants grown in standard conditions were transferred to a light intensity of 950 μmol photons m^-2^ s^-1^ at 8°C. After 1 day, some bleaching appeared particularly in well-expanded and old leaves and damage intensity increased until the 4th day of treatment. Interestingly, a differential behavior was observed: Wt and Trx f^+^ exhibited much larger bleached leaf areas compared to Trx m^+^ plants, which displayed damage limited to leaf edges (**Figure 6A**). To further investigate the responses of tobacco plants to photooxidative stress conditions, we measured maximal photosystem II efficiency, using the chlorophyll fluorescence parameter, *F*_v_/*F*_m_, which constitutes a sensitive indicator of photosynthetic performance and reveals whether photosynthetic structures are damaged (Maxwell and Johnson, 2000). As previously observed in control conditions (**Figure 2**), Wt and Trx f^+^ plants displayed close *F*_v_/*F*_m_ values, 0.785 and 0.754, respectively, whereas this value was already substantially decreased in Trx m^+^ plants (0.691; **Figure 6B**). *F*_v_/*F*_m_ measurements in plants exposed to photooxidative treatment for a period of 5 days revealed a strong decrease in PSII photosynthetic efficiency in Wt and Trx f^+^ plants since the recorded values were reduced by *ca.* 65 and 70% (0.272 and 0.233, respectively). In contrast, the decrease observed in Trx m^+^ plants was much less pronounced, the mean *F*_v_/*F*_m_ value measured in this line being reduced by *ca.* 45% (0.371). These data reveal that photosynthetic efficiency is less impaired in Trx m^+^ plants exposed to photooxidative treatment and are consistent with the visual observations of limited leaf bleaching in this line.

### TOLERANCE TO METHYL VIOLOGEN OF TOBACCO PLANTS OVEREXPRESSING Trx f OR Trx m

**FIGURE 7:**
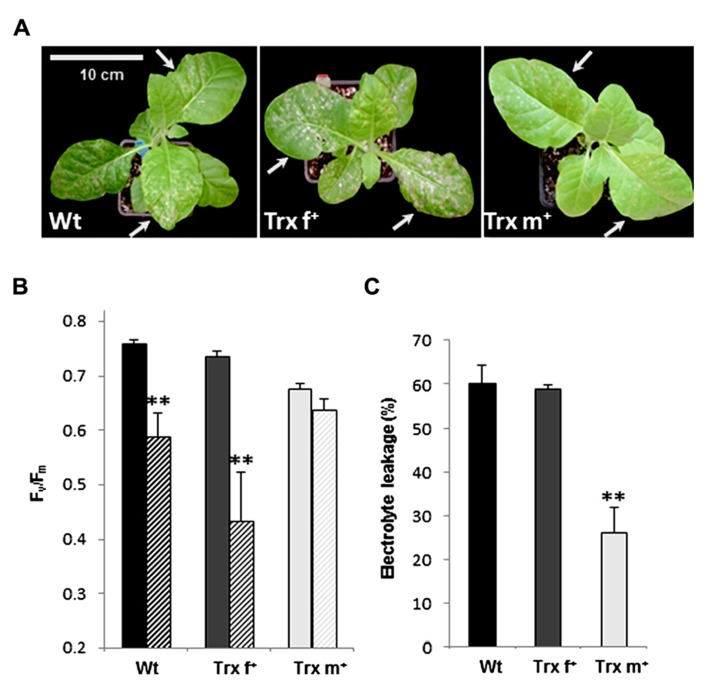
**Tolerance to methyl viologen of transplastomic tobacco plants overexpressing Trx f or Trx m.** 40-day old tobacco plants were sprayed with 30 μM MV and grown in a controlled growth chamber as described in Section “Materials and Methods.” **(A)** Visual symptoms 2 days after treatment. Photographs show representative plants subjected to the treatment. **(B)** Maximal photosystem II photochemical efficiency in tobacco plants. *F*_v_/*F*_m_ values were measured in plants grown in control conditions (full bars) and in plants subjected to MV treatment for 5 h (dashed bars). Data are mean values ± SD from six plants per genotype. Measurements were performed on two leaves (indicated by arrows in panel **A**) per plant. **Value significantly different from the value measured in relative non-treated plants with *p* < 0.01 (*t*-test). **(C)** Electrolyte leakage measured on tobacco leaf disks after incubation on 1 μM MV for 14 h at 600 μmol photons m^-2^ s^-1^. Results were expressed as the percentage of total electrolytes released after treatment relative to that of disks incubated on water under the same conditions. Data are mean values ± SD from three assays. Wt, wild-type; Trx f^+^ and Trx m^+^, plants overexpressing Trx f or Trx m, respectively. **Significantly different from Wt with *p* < 0.01 (*t*-test).

Trx overexpressing plants were also evaluated for protection against damage induced by MV, a redox-cycling herbicide that generates superoxide radicals by accepting electrons from PSI and transferring them to oxygen (Babbs et al., 1989). MV-mediated oxidative damage was assessed in whole plants sprayed with MV, and visual symptoms were registered 2 days following treatment. Wt and Trx f^+^ plants were severely affected by MV treatment, whereas necrotic lesions were more limited in Trx m^+^ plants (**Figure 7A** and data not shown). Maximal PSII efficiency (*F*_v_/*F*_m_) was measured 5 h after MV treatment. Similarly to the results reported above for photooxidative damage, PSII photosynthetic efficiency was significantly impaired in Wt and Trx f^+^ plants (*F*_v_/*F*_m_ values reduced by *ca.* 20 and 40%, respectively), whereas almost no change was observed in Trx m^+^ plants (**Figure 7B**). To further evaluate the tolerance of transplastomic tobacco plants to MV, membrane damage was estimated by measuring ion release from control and treated leaf disks. Following incubation on MV, the level of electrolyte leakage from Trx f^+^ disks was relatively high and comparable to that of Wt (*ca.* 60% of the total electrolyte content, **Figure 7C**). In contrast, plants overexpressing Trx m displayed a much lower level of ion release (25%). Altogether, these results signify that overexpression of Trx m, but not of Trx f, increases tolerance to MV.

## DISCUSSION

### SPECIFICITY OF Trx m FUNCTION

In contrast to other organisms, where only two or three multifunctional Trx isoforms are present (Ritz et al., 2000; Garrido and Grant, 2002), plants display a great diversity of Trxs with physiological functions remaining elusive for many of them. Our work based on a genetic strategy contributes to decipher the role of Trx m *in planta*. Clear differences have been observed between plants overexpressing either Trx m or Trx f. Trx m^+^ plants exhibit reduced growth and impaired photosynthesis (**Figures 1,2**), but increased tolerance to oxidative treatments (**Figures 6,7**). In comparison, Trx f^+^ plants are almost undistinguishable from Wt, in full agreement with previous data (Sanz-Barrio et al., 2013) and others gained in *Arabidopsis* mutants knockout for Trx f (Thormählen et al., 2013) and in Trx f RNAi pea plants (Luo et al., 2012). Consistently, in heterologous complementation assays in yeast, *Arabidopsis* Trx m, but not Trx f, confers tolerance to oxidative stress (Issakidis-Bourguet et al., 2001). Taken together, these reports indicate that Trxs f and m fulfill distinct and non-overlapping physiological functions very likely through a marked specificity towards their targets* in planta*.

In the last years, knowledge has been acquired about the essential roles of some plant Trxs, particularly plastidial isoforms. Some are critical such as Trx z which is needed for plastid development and seedling viability (Arsova et al., 2010). NTRC-KO *Arabidopsis* plants display pale phenotype, impaired photosynthesis and sensitivity to oxidative stress, high temperature and prolonged darkness (Pérez-Ruiz et al., 2006; Lepistö et al., 2009; Chae et al., 2013). Other plastidial Trxs play more specialized functions such as CDSP32, which prevents oxidative damage during environmental constraints (Broin et al., 2002; Rey et al., 2005) and Trx y2, which maintains growth under high light conditions (Laugier et al., 2013). In other respects, cytosolic Trx h1 participates in responses to salt treatment (Zhang et al., 2011) and Trx h5 in sensitivity to a fungal toxin (Sweat and Wolpert, 2007). Our present work reveals that Trx m fulfills essential functions in photosynthetic processes and in stress tolerance. Noteworthy, down-regulation of Trx m gene expression in rice also leads to impaired growth and reduced chlorophyll content in control conditions (Chi et al., 2008). This further argues for a function of Trx m as a central actor controlling photosynthesis. However, no obvious phenotype was recorded in *Arabidopsis* mutants deficient for either Trx m1 or Trx m4 (Courteille et al., 2013; Laugier et al., 2013). Since these Trxs share, with Trx m2, very similar sequence and biochemical properties (Collin et al., 2003), we hypothesize that the three isoforms could play overlapping functions in *Arabidopsis*.

### PHENOTYPE OF Trx m^+^ TOBACCO UNDER CONTROL CONDITIONS

Compared to Wt and Trx f^+^ plants, Trx m^+^ plants grown in control conditions display modified chlorophyll composition and impaired PSII activity (**Figure 2**; **Table 1**). Trx m overexpression in tobacco (**Table 1**), like Trx m deficiency in rice (Chi et al., 2008), leads to lower chlorophyll content and increased Chl *a*/Chl *b* ratio. Luo et al. (2012) showed that pea plants silenced for both genes encoding Trxs f and m exhibit reduced chlorophyll content and a much higher level of the oxidized form of CHLI, a subunit of magnesium chelatase, an enzyme essential for chlorophyll biosynthesis and regulated by Trxs (Ikegami et al., 2007). Moreover, Luo et al. (2012) reported that the expression of numerous genes involved in tetrapyrole biosynthesis was also strongly altered in these pea plants. Therefore, we can hypothesize that expression and redox activation of enzymes participating in chlorophyll synthesis are modified in Trx m^+^ tobacco due to the large Trx excess, leading to change in pigment content. In other respects, one LHCII protein has been identified as a Trx target in spinach thylakoid membranes (Balmer et al., 2006). Interestingly in Trx m^+^ plants, we recorded a much lower content in Chl *b* (**Table 1**), which is more specifically associated with LHC proteins, and preliminary proteomic analyses on transplastomic lines indicate that LHCII proteins are less abundant compared to Wt and Trx f^+^ (unpublished data). All these data prompt us to propose that Trx m might be involved in the regulation of the light capture process via the control of the abundance and/or redox status of LHC proteins. We previously reported that Trx m^+^ plants are characterized by the absence of cyclic electron transfer via the NDH pathway (Courteille et al., 2013). Thus, the suppression of this pathway might modify the NADPH/ATP ratio within plastids and finally impair the whole photosynthetic process. This hypothesis is not supported by the fact that tobacco plants knockout for the whole NDH complex do not exhibit any noticeable phenotype in control conditions (Horváth et al., 2000). Nonetheless, as the other cyclic electron pathway via the proton gradient regulation (PGR) complex is also negatively regulated by Trx m4 in *in vitro* experiments (Courteille et al., 2013), we cannot exclude that inhibition of both cyclic NDH and PGR pathways due to high Trx m abundance results in substantial impairment of photosynthetic processes. In other respects, in control conditions no obvious difference between the two transplastomic lines has been noticed regarding the activities in leaf extracts of two enzymes involved in carbon metabolism, NADP-MDH and NADP-GAPDH (**Figure 3**). Based on all these data, we conclude that the phenotype of Trx m^+^ plants is probably not linked to changes in carbon metabolism, but more likely to modifications in photochemical processes from light capture to electron transfer.

Interestingly, plants overexpressing Trx m display reduced contents in ascorbate and GSH (*ca. *-20% compared to Wt and Trx f^+^ plants), with no modification in redox status (**Figure 5**). This reveals that Trx m exerts a specific control on the amount of these compounds through mechanisms remaining to be delineated. Regarding AsA, redox regulation of enzymes involved in biosynthesis and regeneration pathways could account for the modified content. Indeed, dehydroascorbate reductase (DHAR), the enzyme regenerating AsA from its oxidized form, has been identified as a Trx target in several reports (Marchand et al., 2004; Hägglund et al., 2008; Montrichard et al., 2009) and reduction of DHAR by Trx is known to activate the enzyme (Dixon et al., 2002). Consequently, overexpression of Trx m in tobacco might trigger activation of DHAR and lead to increased AsA recycling, thus explaining the need for a reduced pool of AsA in Trx m^+^ tobacco. But note that in tobacco plants overexpressing DHAR, increased AsA recycling is associated with increased AsA content (Chen et al., 2003). Currently, there is no evidence for a possible redox regulation of enzymes involved in GSH metabolism (Montrichard et al., 2009). It is worth mentioning that the increase in AsA content due to DHAR overexpression in tobacco plants is accompanied by a GSH pool increased in the same range (Chen et al., 2003). Further studies are needed to investigate whether the decreased GSH content in Trx m^+^ plants is linked to modified AsA level or is a direct consequence of Trx overexpression. Whether the modified GSH and AsA pools in Trx m^+^ plants lead to the observed growth and photosynthesis phenotype remains also unclear. Nonetheless, these data unveil a new role for Trx m in plant cell redox homeostasis through regulation of the content in major soluble antioxidants.

### PHENOTYPE OF Trx m^+^ TOBACCO UNDER OXIDATIVE TREATMENTS

Compared to Wt and to Trx f^+^, Trx m^+^ plants are more tolerant to conditions generating oxidative damage, induced by either MV or high light combined to low temperature (**Figures 6,7**), revealing a role of Trx m in the protection of plastidial structures. Accordingly, the abundance of Trx m1 is strongly up-regulated in cold-stressed *Arabidopsis* plants and this Trx has been proposed to preserve photosynthetic apparatus (Goulas et al., 2006). The tolerance of Trx m^+^ plants is nonetheless rather puzzling when taking into consideration their reduced chlorophyll content (by more than 40% for Chl *b*) since an *Arabidopsis* mutant lacking Chl *b* is much more sensitive to photooxidative treatments, partly due to increased single oxygen production (Dall’Osto et al., 2010). Thus, the tolerance of Trx m^+^ tobacco plants is not directly linked to chlorophyll content, but to other mechanisms also counterbalancing the deleterious effects due to Chl *b* shortage. Based on our data, we presume that neither NPQ, nor soluble antioxidants constitute primary determinants in this stress tolerance. Indeed, in Trx m^+^ plants, NPQ is not modified (**Figure 2**) and both AsA and GSH pools are reduced by *ca.* 20% (**Figure 5**), with no change in redox status. In numerous cases, there is a positive correlation between stress tolerance level and AsA content. For instance, an *Arabidopsis* mutant deficient in AsA is highly sensitive to environmental constraints (Conklin et al., 1996) and tobacco plants with increased AsA content are more tolerant to high light (Chen and Gallie, 2008). Regarding GSH, as reviewed very recently (Zagorchev et al., 2013), complex and contradictory data have been reported since tobacco plants with decreased GSH reductase activity are more sensitive to oxidative stress (Ding et al., 2009), but plants displaying elevated GSH biosynthesis capacity show high sensitivity to light (Creissen et al., 1999).

Trx m^+^ plants display reduced PSII activity in control conditions (**Figure 2**), and surprisingly this activity is much less affected under oxidative conditions compared to Wt (**Figures 6,7**). Exposure to environmental constraints generally impairs PSII at the D1 protein level due to increased production of reactive oxygen species and inhibits PSII repair via the suppression of the synthesis of this subunit (Takahashi and Murata, 2008). Thus we can speculate that the excess of Trx m is associated with preservation of PSII structures in stress conditions. In connection with this hypothesis, it is worth mentioning that Trx has been proposed to regulate D1 synthesis as a function of light level (Danon and Mayfield, 1994).

Finally, we can hypothesize that the tolerance of Trx m^+^ plants results from direct prevention by Trx m of damage in the photosynthetic apparatus. We recently reported that both plastidial Trxs f and m are able to form oligomers possessing chaperone-like properties (Sanz-Barrio et al., 2012). However, since there is a differential behavior of Trx m^+^ and f^+^ plants exposed to oxidative treatments, it appears unlikely that such a function could specifically account for the phenotype of Trx m^+^ plants. In other respects, their stress tolerance could originate also from increased electron supply to Trx targets participating in antioxidant mechanisms, thus improving the plant capacity to adapt to challenging environmental conditions. Prxs are not likely involved in such a process since no noticeable difference was observed in abundance and/or redox status of the main plastidial Prxs in Trx m^+^ plants (**Figure 4**). In contrast, we observed a twice higher MSR enzymatic capacity in Trx m^+^ plants than in Trx f^+^ and Wt plants with no change in protein abundance (**Figure 4**). This signifies that Trx m very likely constitutes a physiological electron donor to MSRs. This is consistent with our previous work showing that while MSR capacity is slightly lowered in *Arabidopsis* plants deficient for Trx f, it is significantly reduced in mutants lacking various Trx m types (Laugier et al., 2013). MSRs are key enzymes repairing oxidized proteins and possibly scavenging ROS via MetO (Moskovitz et al., 1997). In plants, they are involved in the protection against environmental constraints (Romero et al., 2004; Laugier et al., 2010) and in seed longevity (Châtelain et al., 2013). Altogether, these data give high credence for a role of the Trx m/MSR system in the tolerance of Trx m^+^ plants to oxidative treatments.

To conclude, the complex phenotype of transplastomic tobacco Trx m^+^ plants indicates that this Trx is very likely a central actor in plant cell redox homeostasis. In contrast to other types like f, the m type could regulate *in planta* numerous redox-based processes in photosynthesis and antioxidant responses. Further investigations, based for instance on co-immunoprecipitation methods to isolate Trx m partners in plant extracts (Rey et al., 2005), are needed to unveil the target proteins and the mechanisms underlying the physiological function(s) of this Trx type.

## Conflict of Interest Statement

The authors declare that the research was conducted in the absence of any commercial or financial relationships that could be construed as a potential conflict of interest.
